# Effects of bupivacaine or levobupivacaine on cerebral oxygenation during spinal anesthesia in elderly patients undergoing orthopedic surgery for hip fracture: a randomized controlled trial

**DOI:** 10.1186/s12871-019-0682-1

**Published:** 2019-01-31

**Authors:** Roser Vives, Diana Fernandez-Galinski, Francisca Gordo, Alberto Izquierdo, Joan C. Oliva, Carmen Colilles, Caridad Pontes

**Affiliations:** 1 Departament de Farmacologia, de Terapèutica i de Toxicologia, UAB, Clinical Pharmacology Unit, Parc Taulí Hospital Universitari, Institut d’Investigació i Innovació Parc Taulí I3PT, Universitat Autònoma de Barcelona, Parc Taulí 1, 08028 Sabadell (Barcelona), Spain; 2 Anesthesiology Department, Parc Taulí Hospital Universitari, Institut d’Investigació i Innovació Parc Taulí I3PT, Universitat Autònoma de Barcelona, Parc Taulí 1, 08028 Sabadell (Barcelona), Spain; 3 Statistics Unit, Parc Taulí Hospital Universitari, Institut d’Investigació i Innovació Parc Taulí I3PT, Universitat Autònoma de Barcelona, Parc Taulí 1, 08028 Sabadell (Barcelona), Spain

**Keywords:** Spinal anesthesia, Regional cerebral oxygen saturation, Bupivacaine, Levobupivacaine

## Abstract

**Background:**

Bupivacaine and levobupivacaine have similar pharmacokinetic and pharmacodynamic characteristics, and are used regularly in spinal anesthesia. Whether potential differences in their hemodynamic and anesthetic profiles could determine a differential risk of complications in elderly subjects, is controversial. The main objective was to compare the effects of intrathecally administered levobupivacaine (LB) versus bupivacaine (B), on regional cerebral O_2_ saturation during spinal anesthesia, cognitive status and neurological complications in elderly patients undergoing surgery for hip fracture.

**Methods:**

This was a randomized, controlled, single blind study. 58 patients aged 70 or older undergoing surgery for hip fracture with spinal anesthesia were allocated with a 1:1 ratio to receive LB or B, combined with fentanyl 15 μg, by intrathecal route. The primary outcome was the proportion of intraoperative time with regional cerebral desaturation (≥20% reduction in regional cerebral oxygen saturation from baseline), monitored by near –infrared spectroscopy. Secondary endpoints included hemodynamic parameters, level of sensory and motor block, changes in Short Portable Mental Status Questionnaire (SPMSQ), and neurological complications.

**Results:**

The mean percentage of intraoperative time with desaturation in the B group was 6.1% (SD: 17.5) and 4.7% (SD: 11.9) in the left and right hemisphere respectively; in the LB group the mean was 4.8% (SD: 11.4) in the left hemisphere and 2.4% (SD: 8.3) in the right one. No statistically significant differences were found between treatment groups. The level of sensory block at the start of surgery was lower for LB than for B (Th10 vs Th8, p:0.047) and motor block at 15 min was lower for LB (2.5 vs 3, p:0.009). No differences in postoperative SPMSQ were observed. Neurological complications such as confusional state, agitation or disorientation were reported in 50% of patients in the B group and 21.4% of patients in the LB group, *p* = 0.05.

**Conclusions:**

No statistically significant differences in regional cerebral oxygen saturation or hemodynamic parameters were observed between both treatment groups. Bupivacaine and levobupivacaine differed in sensory and motor block achieved. While no differences were observed in cognitive impairment measured by the SPMSQ between treatment groups neurological complications reported by the physician were more frequent with bupivacaine.

**Trial registration:**

European Union Clinical Trials Register (EudraCT 2013–000846 -20) (April 9th, 2013).

ClinicalTrials.gov (NCT01960543) (September 23rd, 2013).

## Background

Decline in cognitive function in elderly patients during hospitalization has been, in part, attributed to sudden changes in the usual environment of the patient, but also, to the acute morbidity or exacerbation of pre-existing conditions. Geriatric patients suffering a hip fracture who undergo surgery are an especially frail population, because of concomitant morbidity, polypharmacy and bleeding. A multi-factorial intervention aimed to preserve cognitive function in this population during the acute hospital stay has shown good results [1]. Spinal anesthesia would be part of this multi-factorial interventions [1], although the evidence on the differential effects of spinal and general anesthesia on cognition is controversial. Shorter length of hospitalization, which could result in fewer complications, has been described for regional anesthesia, but it does not reduce mortality [2].

Intraoperative hypotension is an undesirable and frequent effect of spinal anesthesia, which may complicate a pre-existing coronary heart disease, worsen previous mental impairment or precipitate a cerebral stroke. Different strategies to maintain the hemodynamic stability of the patient include the choice of anesthetic drugs with the most favorable hemodynamic profile, and use of reduced doses of local anesthetic [3]. Racemic bupivacaine (B) and levobupivacaine (LB) are closely related local anesthetic agents; controversial reports suggest that they have different anesthetic profiles, with B inducing higher blockade, more hypotension, and consequently, potentially more cerebral desaturation [4, 5].

Clinical monitoring of CNS is rarely performed during spinal anesthesia. Instead, indirect parameters such as arterial pressure, heart rate and arterial O_2_ saturation are registered [6]. Regional cerebral oxygen saturation (rCSO_2_) through near –infrared spectroscopy, a non invasive monitoring that reflects a balance between supply and consumption of O_2_ to the brain, has been proposed as a tool that may provide relevant information on CNS ischemic stress, and may be potentially useful to guide the clinical management of the patient during spinal anesthesia [7]. Thus, rCSO_2_ might also be useful to assess the CNS consequences of the differences in the hemodynamic and anesthetic profile between two different anesthetic agents used in spinal anesthesia.

Currently, both B and LB are used interchangeably for spinal anesthesia in our hospital. Our working hypothesis was that cerebral desaturation would be higher in patients treated with B than in those receiving LB, and that this could be related to worse neurological outcomes. In order to test the hypothesis, we designed the present pragmatic randomized clinical trial to compare the effect of B and LB on regional cerebral oxygen saturation, during spinal anesthesia in elderly patients undergoing surgery for hip fracture repair, both administered by spinal route and according to routine clinical practice. We also compared the effects of both anesthetics on postoperative cognitive status and incidence of nervous system related adverse events.

## Methods

### Design

This was a randomized, controlled, single-center, single blind trial conducted at Hospital de Sabadell. The protocol was approved by the Ethics Committee of the Centre on March 2013 (*CEIC Corporació Sanitaria Parc Taulí, Sabadell,* reference number 2013/015). The study was registered at the European Union Clinical Trials Registry (EudraCT 2013–000846 -20) on April 9th, 2013 and at ClinicalTrials.gov (NCT01960543) on September 23rd, 2013. All patients received comprehensive information by the investigator on the study objectives, procedures and risks and signed the informed consent form before inclusion. Informed consent in front of a witness was allowed for patients unable to write or read due to physical impairment but with preserved cognitive function.

### Patients

Patients aged 70 years and older requiring surgery for hip fracture repair that were suitable to receive spinal block,were included. Prior to inclusion, patients underwent cognitive examination by means of the Short Portable Mental Status Questionnaire (SPMSQ) [8, 9] and those with low scores (≥ 8 failures if elementary school, ≥ 7 failures if higher education), were excluded. Patients were excluded if they had any contraindication to B or LB (hypersensitivity to amide local anesthetics, severe aortic stenosis, heart failure or coagulopathies) or if they refused the regional anesthetic technique.

### Methodology

A random list was generated by the clinical pharmacology unit using Winpepi V2.67 [10]. Sequentially numbered sealed opaque envelopes were produced containing the treatment identity for each patient. The list and a copy of the codes were kept in a file not accessible to the study team. Inclusion was confirmed by the anesthetist, who opened the first available sequentially numbered sealed code to randomize the patient. The study was blind for patients and for the anesthesiologists who evaluated the postoperative SPMSQ questionnaire.

Patients were not pre-medicated. Antihypertensive drugs were discontinued on the day of surgery and restarted at 48 h after surgery. Patients were randomly allocated (ratio 1:1) to receive treatment with racemic bupivacaine (Bupivacaine Braun 0.5%®, B. Braun Medical, S. A, Spain) or levobupivacaine (Chirocane®, Abbvie Spain S.L.U, Spain), both combined with fentanyl 15 μg, by intrathecal route. Both B and LB were isobaric. Spinal anesthesia was administered through a 25 gauge spinal needle using the midline or paramedial approach. The B and LB dose administered was 7 mg if height was < 160 cm and 9 mg if height was ≥160 cm, or when the surgical procedure was a hip prosthesis. Low doses were used with the objective to maintain the hemodynamic stability and minimize intra-operative hypotension. Supplemental oxygen administration was started after spinal injection. As per the protocol, if the anesthesia was not sufficient to initiate surgery, midazolam 1 mg and propofol 30 mg could be administered, and if not effective, general anesthesia was induced and the patient was withdrawn from the study. In the operating room patients received 300 ml of Lactated Ringer’s over 20 min. Electrocardiography, cuff for non-invasive blood pressure measurement, and pulse oximetry (Philips Intellivue MP70 Anesthesia) were used for routine monitoring. Hypotension (systolic blood pressure (SBP) < 100 mmHg) was treated with iv phenylephrine 50–75 μg, and ephedrine 5 mg in case the hypotension did not revert after two doses of phenylephrine. Bradycardia (heart rate (HR) < 50 beats/min) was treated with atropine 0.5–1 mg.

### Outcomes and measurements

Patient characteristics such as ASA physical status, patient’s pathologies and treatments, type of fracture and surgery, SPMSQ and preoperative hemoglobin were recorded. In the operating room, and after spinal anesthesia, the level of sensory block (SB) was evaluated by pinprick, and motor block (MB) by the modified Bromage scale 15 min after the spinal injection, before and at the end of the surgery. The modified Bromage scale is used to measure the intensity of motor block based on the ability of the patients to move their lower extremities (0 = no paralysis, 1 = able to move knees, unable to raise extended legs, 2 = able to flex ankles unable to flex knees, 3 = unable to flex ankles, knees or hips). After surgery, SB was evaluated every 30 min until regression to L5 level.

Regional cerebral oximetry was measured by near–infrared spectroscopy using INVOS 5100 C, 7.00.0014 version (Somanetics Inc., Troy, MI, 48084 - USA). A first measure of rSCO_2_ was performed at entry in the operating room. A second measurement taken 2 min later was considered as the reference baseline value for each hemisphere, and values were recorded throughout the study. Regional cerebral desaturation was defined as a 20% or larger reduction in rCSO_2_ with respect to the ipsilateral baseline value. Routine monitoring of SBP, mean arterial pressure (MAP), HR and O_2_ saturation (O_2_ Sat) monitoring, volume of crystalloid infused, intraoperative use of vasoconstrictor drugs, postoperative hemoglobin and the need for blood transfusions, were also recorded.

The primary outcome measure was the mean proportion of intra-operative time with regional cerebral oxygen desaturation, defined as an rSCO_2_ relative reduction of 20% from baseline value. The area under the curve (AUC) for the intraoperative time below the desaturation threshold was also calculated (AUC rCSO_2_ reduced by ≥20% from baseline). Similar calculations were done using an absolute value of 50% as a cutoff point (AUC rCSO_2_ < 50%). Minimum values (trough rCSO_2_) and time to first saturation drop under the different cut off points were also recorded.

Postoperative cognitive impairment was evaluated after 5 to 7 days, by changes in the SPMSQ scores from baseline. Any adverse events related to nervous system disorders, reported by the responsible physician within 30 days after surgery were also recorded.

### Statistical methods

A sample size of 28 patients per group was calculated assuming an expected clinically relevant difference between treatments of 0.15 or higher on the mean proportion of intraoperative time with desaturation, with a standard deviation (SD) of 0.2, an alpha of 0.05 and 80% power.

Qualitative variables were described by the number of valid values and the frequency and percentage of each category, and compared between groups using Fisher exact tests. Quantitative variables were described by measures of central tendency and dispersion, and compared between groups using a Student T test or Mann-Whitney’s test, as appropriate. A post-hoc bivariate analysis using the above mentioned statistical tests was performed to explore the relationship of selected variables with intraoperative desaturation, postoperative cognitive impairment and neurological complications. Variables showing statistical significance in the bivariate analysis were included in logistic regression multivariate analyses to explore predictors of regional cerebral desaturation, cognitive impairment or neurological complications.

## Results

**Fig. 1:**
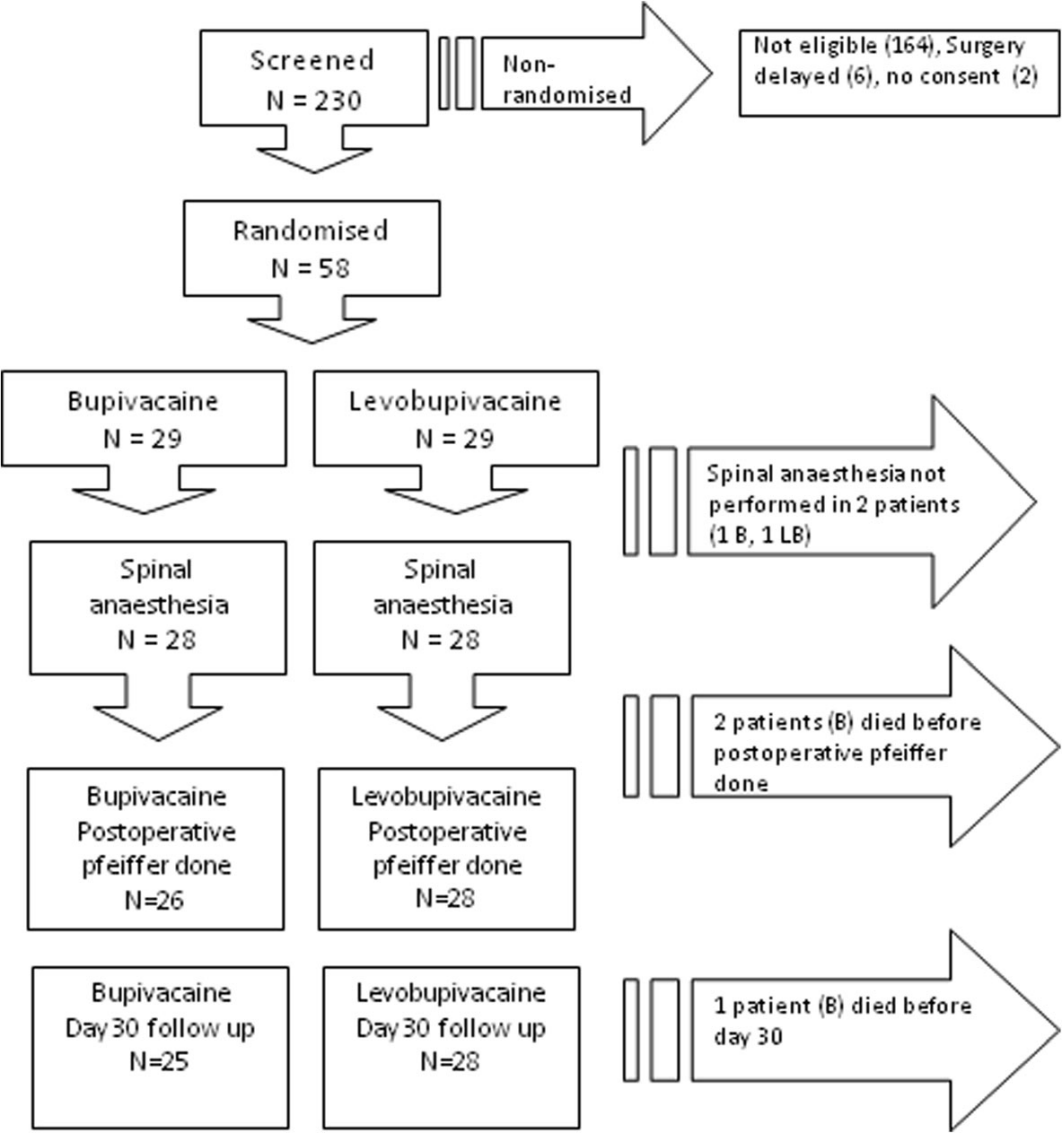
Disposition of patients

From September 2013 to December 2014, 58 patients meeting all the eligibility criteria were randomized. In two patients, the spinal anesthesia was not feasible and they were converted to general anesthesia; they were excluded from the analysis. At the start of surgery, three patients in the LB group needed midazolam + propofol and one patient in each group received midazolam alone. Figure 1 displays the disposition of patients, and Table 1 shows the baseline characteristics of patients.

**Table 1:** Baseline characteristics

*Description of patients*	*Bupivacaine (n = 28)*		*Levobupivacaine (n = 28)*	
	N	%	N	%
Gender				
*Female*	19	67.9	24	85.7
ASA physical status				
*1*	0	0	1	3.6
*2*	2	7.1	9	32.1
*3*	26	92.9	18	64.3
Education				
*Elementary school*	26	92.9	27	96.4
*High school/University degree*	2	7.1	1	3.6
Concomitant diseases				
*Hypertension*	18	64.3	18	64.3
*Diabetes Mellitus*	13	46.4	6	21.4
*Dyslipidemia*	7	25.0	8	28.6
*Arrhythmia*	7	25.0	5	17.9
*Renal Failure*	7	25.0	3	10.7
*Cerebrovascular disease*	1	4.2	4	16.7
*Depression*	1	3.6	6	21.4
Concomitant treatments				
*Diuretic*	14	50.0	11	39.3
*ACE inhibitors*	10	35.7	7	25.0
*AR Blockers*	6	21.4	8	28.6
*Calcium antagonists*	7	25.0	4	14.3
*Beta-blockers*	5	17.9	6	21.4
*CNS drugs*	19	67.9	25	89.3
*Benzodiazepines*	11	39.3	11	39.3
*Antidepressants*	8	28.6	12	42.9
Fracture type				
*Pertrochanteric*	17	60.7	11	39.3
*Subtrochanteric*	1	3.6	5	17.9
*Femoral neck*	8	28.6	7	25
*Periprosthetic*	1	3.6	2	7.1
*Supracondylar*	1	3.6	1	3.6
*Distal diaphyseal*	0	0	2	7.1
Surgery type				
*Intramedullary internal fixation*	16	57.1	14	50.0
*Extramedullary internal fixation*	5	17.9	8	28.6
*Total hip arthroplasty*	3	10.7	1	3.6
*Cemented arthroplasty*	4	14.3	3	10.7
*Cementless hemiarthroplasty*	0	0	2	7.1
Local anesthetic doses				
*7 mg*	8	28.6	7	25.0
*9 mg*	20	71.4	21	75.0
Right rCSO2 lower than Left rCSO2	14	50.0	11	39.3
	Mean (SD)	n	Mean (SD)	N
Age (years)	83.1 (5.5)	28	83.7 (6)	28
Weight (Kg)	66 (10.3)	28	67.6 (11.7)	28
Height (cm)	161 (7)	28	159.1 (6.3)	28
Baseline hemoglobin (g/L)	112.6 (19)	28	116.2 (18.1)	28
Duration on surgical intervention (min)	60.6 (24.7)	28	78.9 (35.5)	28
Baseline SBP (mmHg)	150.8 (24.7)	28	148.7 (21.8)	28
Baseline MAP (mmHg)	87.1 (14.2)	27	87.8 (14.4)	27
Baseline HR (bpm)	78.7 (11.7)	28	81.5 (11.5)	28
Baseline oxygen saturation (%)	92.4 (6.6)	28	93.9 (2.7)	28
Baseline rCSO2 (%) Left	59.8 (7.8)	28	59.7 (10.4)	28
Baseline rCSO2 (%) Right	58.8 (9.1)	28	60.1 (11.2)	28

*ASA* American Society of anesthesiologists, *bpm* beats per minute, *HR* heart rate, *MBP* mean blood pressure, *SBP* systolic blood pressure, *SD* standard deviation, *rCSO*_*2*_ regional cerebral oxygen saturation

Most patients (57.1%) had at least 3 comorbidities at hospital admission. Table 1 shows the most frequent concomitant diseases per treatment group. 78.3% of patients were taking 3 or more different medications on a regular basis, and 53.6% more than 4. Table 1 shows the treatments most frequently used by patients per treatment group. The majority of patients were operated on within 2 days after admission, and the time from admission to surgery was evenly distributed in both treatment groups.

No significant differences were observed between treatment groups in the proportion of intraoperative time with desaturation, nor in any of the secondary variables based on the measurement of rCSO_2_ (Table 2). Overall, 46% of patients (13 B; 13 LB) presented a relative reduction in rCSO_2_ > 20% from baseline in either one or both hemispheres; considering absolute values of rCSO_2_ below 50, 64.3% (B) and 53.6% (LB) showed desaturation at some time point. Hemodynamic parameters (SBP, MAP and HR) were comparable between the two groups, as well as the need for vasoconstrictor drugs, although a trend to a lower SBP for patients was observed in B group (Table 3). The anesthetic profile for each treatment group is shown in Table 3. Intraoperative red blood cell transfusion was required in 3 patients in the B group and in 4 patients in the LB group. The mean (SD) volume of crystalloid infused were 605.4 (321.6) in the B group and 589.3 (240.5) mL in the LB group (*p* = 0.83).

**Table 2:** Intraoperative regional cerebral oxygen saturation with bupivacaine and levobupivacaine

	All patients			Patients with any desaturation		
	B	LB		B	LB	
Desaturation > 20% below baseline						
Left hemisphere	(*n* = 28)	(*n* = 28)		(*n* = 9)	(*n* = 12)	
AUC (min %)	14.93 (42.60)	12.43 (30.86)	ns	69.67 (72.28)	34.80 (44.63)	ns
Time below 20% from baseline (min)	5.57 (14.47)	5.21 (13.14)	ns	17.36 (21.84)	12.14 (18.21)	ns
% of time with desaturation	6.07 (17.47)	4.82 (11.40)	ns	18.9 (27.52)	11.25 (15.49)	ns
Time to first deep (min)	–	–	ns	23.67 (22.31)	32.66 (30.27)	ns
Right hemisphere	(*n* = 28)	(*n* = 28)	ns	(*n* = 9)	(*n* = 10)	ns
AUC (min %)	14.32 (35.34)	4.32 (14.98)	ns	57.29 (52.43)	15.3 (26.05)	ns
Time below 20% from baseline (min)	4.74 (11.87)	2.44 (8.30)	ns	14.75 (17.58)	6.84 (13.15)	ns
% of time with desaturation	4.01 (9.33)	2.16 (7.08)	ns	14.02 (13.24)	6.04 (11.15)	ns
Time to first deep (min)	–	–	–	29.28 (25.65)	45.97 (22.56)	ns
Desaturation < 50% (absolute values)						
Left hemisphere	(*n* = 28)	(*n* = 28)		(*n* = 15)	(*n* = 13)	
AUC (min %)	53.07 (108.82)	158.29 (440.69)	ns	114.31 (138.06)	340.92 (607.88)	ns
Time below 20% from baseline (min)	12.82 (24.37)	16.59 (36.85)	ns	23.93 (29.32)	35.75 (48.11)	ns
% of time with desaturation	11.62 (19.38)	13.65 (30.69)	ns	21.68 (22.24)	29.41 (40.20)	ns
Time to first deep (min)	–	–	ns	20.71 (30.77)	30.57 (35.09)	ns
Right hemisphere	(*n* = 28)	(*n* = 28)	ns	(*n* = 17)	(*n* = 13)	ns
AUC (min %)	77.54 (152.84)	134.14 (422.71)	ns	127.71 (180.6)	288.9 (594.63)	ns
Time below 20% from baseline (min)	15.84 (30.14)	13.32 (31.68)	ns	26.09 (35.34)	28.68 (42.20)	ns
% of time with desaturation	13.08 (23.03)	11.12 (26.55)	ns	21.54 (26,49)	23.94 (35.40)	ns
Time to first deep (min)	–	–	–	28.80 (34.42)	19.12 (27.98)	ns
Lowest values rCSO2 (%)						
	(*n* = 28)	(*n* = 28)				
Left hemisphere	49.46 (8.59)	48.32 (11.71)	ns			
Right hemisphere	48.18 (10.60)	49.86 (11.87)	ns			

*B* Bupivacaine, *LB* Levobupivacaine, *AUC* area under the curve, *rCSO2* regional cerebral oxygen saturation, *ns* not significant *p* value. Values expressed as mean (Standard Deviation) for all patients of the treatment group and for patients who presented desaturation of the measured hemisphere

**Table 3:** Anesthetic and intraoperative hemodynamic profile of bupivacaine and levobupivacaine

*Anesthetic profile*	*N*	*Bupivacaine*	*N*	*Levobupivacaine*	
Sensory Block (*median – range*)					
15 min after SI	25	Th9 (Th4-Th12)	20	Th11 (Th5-TL2)	p: 0.14
Start of surgery	23	Th8 (Th4-T12)	22	Th10 (Th5 – L2)	p: 0.047*
End of surgery	24	Th11 (Th5-L5)	21	Th11 (Th7- L5)	p: 0.61
Duration of SB (min) (mean ± SD)	24	178.25 ± 44.89	22	175.18 ± 42.53	p: 0.81
Motor Block (*median – range*)					
15 min after SI	25	3 (2–3)	20	2.5 (1–3)	p: 0.009*
Start of surgery	24	3 (2–3)	22	3 (1–3)	p: 0.057
End of surgery	25	3 (0–3)	21	1 (0–3)	p: 0.21
Duration of MB (min) (mean ± SD)	25	157.04 ± 41.71	23	141.65 ± 56.02	p: 0.29
Time (min) (Mean ± SD)					
SI to Start of surgery	25	21.32 ± 6.80	23	19.78 ± 7.45	p: 0.46
SI to End of surgery	25	80.75 ± 27.88	23	97.67 ± 35.97	p: 0.055
Surgery	28	60 ± 24.6	28	78.6 ± 35.4	p: 0.03*
Hemodynamic profile					
Patients with hypotension (n/%)	28	19 / 67.8%	28	20 / 71.4%	p: 0.99
Minimum values (Mean ± SD)					
Lowest SBP (mmHg)	28	84.0 ± 26.43	28	96.03 ± 21.31	p: 0.06
Lowest MAP (mmHg)	28	53.03 ± 14.11	28	56.96 ± 14.20	p: 0.31
Lowest HR (beats/min)	28	65.57 ± 10.66	28	65.57 ± 12.68	p: 0.52
Use of Vasoconstrictor drugs					
*Phenylephrine* n° patients / mean dose (ug) ± SD	28	17 / 258.82 ± 181.12	28	16 / 239.06 ± 153.83	p: 0.73
*Ephedrine* n° patients / mean dose (mg) ± SD	28	6 / 11.83 ± 5.19	28	7 / 15.57 ± 13.62	p: 0.52
*Atropine 0.5 mg (n/%)*	28	0/0.0%	28	3/10.7%	p:0.24

*SB* sensory block, *MB* motor block, *SI* spinal injection, *SBP* systolic blood pressure, *MAP* mean arterial pressure, *HR* heart rate. *SD* standard deviation. Motor Block (0 = no paralysis, 1 = able to move knees, unable to raise extended legs, 2 = able to flex ankles unable to flex knees, 3 = unable to flex ankles, knees or hips)

Postoperative SPMSQ scores were overall better than those observed at baseline (Table 4), and no statistically significant differences were detected between treatment groups. Worsening in the SPMSQ score after surgery was observed in 23.1% of patients in the B group, and in 28.6% of patients in the LB group. No differences in changes from baseline in SPMSQ scores were observed between treatment groups, even after adjusting by baseline SPMSQ scores.

**Table 4:** SPMSQ scores at baseline and after 5–7 days

	Baseline			Post-op		
	B	LB		B	LB	
	(*n* = 28)	(*n* = 28)		(*n* = 26)	(*n* = 28)	
SPMSQ			ns			ns
*0–2 errors*	9 (32.1%)	13 (46.4%)		14 (53.8%)	16 (57.1%)	
*3–4 errors*	12 (42.9%	9 (32.1%)		8 (30.8%)	8 (28.6%)	
*5–7 errors*	7 (25.0%)	6 (21.4%)		4 (15.4%)	3 (10.1%)	
*8 or more errors*	0	0		0	1 (3.7%)	
SPMSQ change, mean (SD)				0.50 (1.96)	0.32 (1.63)	ns

*B* Bupivacaine, *LB* Levobupivacaine, *SPMSQ* Short Portable Mental Status Questionnaire, *SD* standard deviation, ns not significant p value

Three patients from the B group died, and none in the LB group (*p* = 0.24). Reasons were aspiration pneumonia followed by heart failure, worsening of a pre-existing chronic renal failure, and sepsis following a respiratory infection, respectively. None of the deaths were considered related to the study treatment by the investigators.

Neurological complications were reported more often in patients in the B group (14, 50%) compared to the LB group (6, 21.4%), *p* = 0.0496. Confusional state was reported in 10 patients (7 B, 3 LB), agitation in 3 patients (2 B, 1 LB), cognitive impairment in 2 patients (1 B, 1 LB) and disorientation in 2 patients (1 B, 1 LB); three patients experienced agitation and disorientation (1 B), bradypsychia-dysartria (1 B) and encephalopathy related to hypercalcemia (1 B).

Baseline mean hemoglobin values were similar in both treatment groups (112.6 g/L and 116.2 g/L for B and LB respectively), and the drop in hemoglobin post-surgery was comparable in both groups (106.2 g/L for B and 110.4 g/L for LB). Anemia was reported as an adverse event in 80.4% of patients, and was similar for B and LB groups, with 9 and 6 patients in B and LB groups respectively, requiring postoperative transfusion (*p* = 0.546). Urinary infections were reported in 4 and 5 patients, renal failure in 8 and 5 patients, and respiratory infections and/or respiratory failure in 3 and 3 patients in B and LB groups, respectively.

**Fig. 2:**
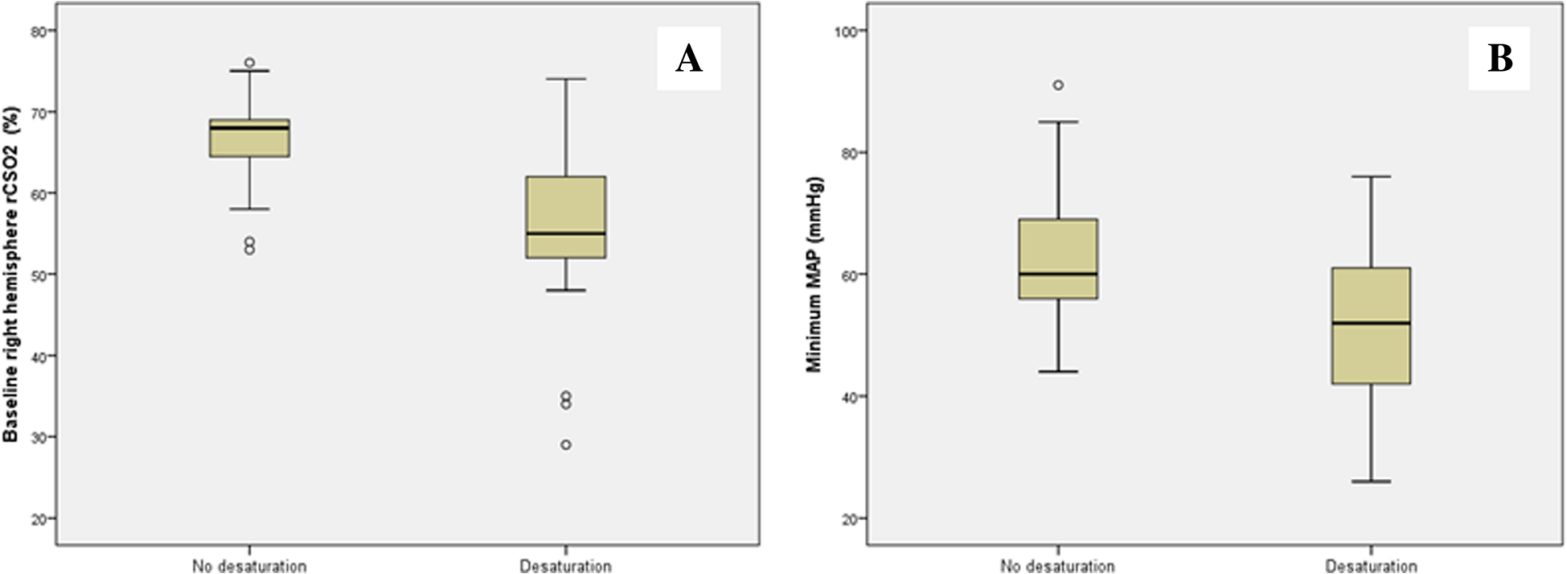
Mean values and dispersion for (A) baseline right hemisphere rCSO_2_ and (B) minimum intra-operative MAP in patients with and without intraoperative desaturation. Footer: rCSO_2_: regional cerebral saturation of oxygen. MAP: Mean arterial pressure

The multivariate exploratory analysis showed that intraoperative desaturation was associated with low rCSO_2_ in the right hemisphere at baseline, so that the risk of desaturation, increased by 61 fold in patients with baseline rCSO_2_ in the lowest quartile, and by 38 fold in those in the second quartile, as referred to the highest rCSO_2_ quartile (Table 5 and Fig. 2). Similarly, subjects in the lower quartile for trough intraoperative MAP had a 29 fold increased risk of desaturation, as compared to the highest quartile (Table 5 and Fig. 2). No variable was found to be significantly predictive of postoperative cognitive impairment, defined as a worsening in the SPMSQ score. In the multivariate analyses, only low SPMSQ scores at baseline predicted neurological complications (OR = 1.58 for each additional incorrect answer in the baseline SPMSQ score, 95%CI: 1.14–2.21).

**Table 5:** Multivariate model for intraoperative desaturation risk

	N	Mean	SD	OR (CI 95%)	*p* value
*rCSO2 –Right* ^*a*^ *(overall)*	*56*	*59.41*	*10.128*		0.020
1st quartile	15	47.53	7.954	61.1 (3.3 to 1127.9)	0.006
2nd quartile	14	55.79	1.626	37.6 (2.2 to 635.2)	0.012
3rd quartile	12	64.00	2.000	2.1 (0.4 to 12.1)	0.429
4th quartile	15	71.00	2.803	(Ref)	
*MAPmin* ^b^ *(overall)*	*54*	*55.00*	*14.17*		0.145
1st quartile	13	36.23	7.33	28.7 (1.4 to 604.8)	0.031
2nd quartile	15	51.80	2.98	9.9 (0.8 to 122.3)	0.074
3rd quartile	13	59.92	1.89	4.3(0.4 to 51.3)	0.244
4th quartile	13	72.54	8.07	(Ref)	
Exp				0.085	0.045

*OR* Odds Ratio, *CI* Confidence interval, *MAP* Mean Arterial Pressure, *SD* Standard Deviation

^a^Baseline regional Cerebral Saturation of Oxygen in Right Hemisphere; ^b^Minimum value for Mean Arterial Blood pressure, intraoperative

## Discussion

Our study found no significant differences between B and LB in any of the endpoints based on regional cerebral oxygen saturation. Cognitive impairment assessment by SPMSQ did not show mental decline for most subjects, but patients receiving B had significantly more reported neurological complications. The population included in our study was a very aged population (mean age of 83.5 years, with 8 (14%) patients aged 90 or older). Patients receiving LB had lower sensory and motor blockades, as expected, and those receiving B had a trend towards lower SBP. This is consistent with the results reported by Erdil et al. comparing B and LB (7.5 mg) in urologic procedures [5] and can be explained by a higher density of LB compared with bupivacaine [11].

rCSO_2_ is determined by a balance between cerebral oxygen supply and demand, and cerebral oxygen supply is related to hemoglobin concentration, arterial O_2_ saturation and blood volume; all of them are factors that can be adversely affected during spinal anesthesia. Some studies comparing spinal versus general anesthesia have shown varying incidences of cerebral desaturation related to the anesthetic technique [12, 13]. During spinal anesthesia in urologic surgery, Nishikawa observed a 10% reduction in rCSO_2_ in patients with higher levels of sensory block (>Th4.7) [14]. Also, in a randomized study in elderly patients undergoing abdominal surgery under general anesthesia, the use of rCSO_2_ monitoring avoided cerebral desaturation and was associated with a reduced incidence of postoperative cognitive decline [6].

We have predefined two cutoff points for assessment of desaturation: a relative change ≥20% from baseline, and an absolute saturation below 50%. We compared the percentage of time that patients spent below these thresholds, and also the lowest values achieved. These variables have not previously been reported for very elderly patients with spinal anesthesia, and in our study, we did not observe any difference between the treatment groups, although differences in the anesthetic profile and rate of complications between B and LB were observed, suggesting that both anesthetic agents are not equivalent in efficacy.

During surgery, we provided tailored prehydration and supplementary oxygen, transfused red blood cells and used vasoconstrictor drugs (mainly phenylephrine) to manage hypotension in every patient presenting SPB measures lower than 100mmHg [15]. Despite intensive management, 46% of patients had at least one drop of rCSO_2_ of 20% below the baseline value, in at least one cerebral hemisphere. Reference values of rCSO_2_ range from 60 to 80% [16]. At baseline our patients had mean rCSO_2_ values around 60%, so that drops below 50% were easily and quickly reached, while reductions of rCSO_2_ ≥ 20% from baseline were less frequent. These findings are consistent with the baseline rCSO_2_ of 63 ± 8% described by Casati et al. for relatively healthy patients aged around 70 [5], and Carlson et al. described that, awake elderly, with marginal rCSO_2_ levels (50–60%), had the greatest changes in rCSO_2_ during sleep that may affect subject’s cognition [17]. Moreover, we observed a wide inter-patient variability in the number and duration of drops below our proposed thresholds of rCSO_2_, especially in subjects already showing some degree of baseline desaturation. Such variability may have compromised the power of the study for the main hypothesis, and may be indicative of either the lack of suitability of rCSO_2_ as a surrogate of cerebral oxygen saturation during spinal anesthesia, or of a choice of thresholds unable to discriminate clinically relevant desaturation. Future studies should consider including a more homogeneous population in terms of rCSO_2_ and the definition of more stringent thresholds for the main variables.

While in some studies intraoperative cerebral desaturation has been found to be significantly associated with an increased risk of cognitive decline [6, 18], a recent review did not show evidence of a relationship between a low rCSO_2_ and neurological complication [19]. Besides, intrasubject differences in rCSO_2_ between L and R during anesthesia has been also been suggested as a risk factor for neurological impairment [20]. In our study, the desaturation variables studied did not prove to be useful as predictors of cognitive decline; however, we found that neurological complications were more frequent with B. Such observation could be due to chance, but could also be due to the trends towards lower values of SBP and MAP in B group than in LB group [21], since a higher sympathetic block may exaggerate hypotension after spinal anesthesia. In our study, no standardized method to assess and report neurological complications was considered, and this variable was only based on the clinical observation of the physicians. This is a limitation of the study and may be the reason for this finding.

Our post-hoc multivariate analyses also found significant associations between preoperative SPMSQ scores and NC, and the risk of intraoperative desaturation was substantial and increasingly higher for patients in the lower quartiles of baseline rCSO_2_ in the right hemisphere. Preoperative SPMSQ has been widely reported in the literature as a risk factor of cognitive decline [1], but low baseline rCSO_2_ in the right hemisphere as a risk factor for desaturation could be further explored as a potential marker of subjects in whom more preemptive hemodynamic management could be indicated. Salazar et al. found that, in patients undergoing total knee replacement, right rCSO_2_ values lower than left rCSO_2_ was a factor associated with the presence of memory decline. We did not explore the asymmetry between left and right hemisphere saturation as a factor associated with cerebral desaturation or cognitive impairment, but our finding that patients with lower right rCSO_2_ values present a higher risk of desaturation may be related to this asymmetry. This finding should be further explored in future studies.

Regarding the assessments of cognitive function based on the SPMSQ, we did not observe differences either, since few patients worsened their scores after surgery. SPMSQ is a test easy to administrate and suitable for very aged patients immobile due to the fracture, who often have forgotten their glasses and who often had poor literacy. However, the first assessment was after a hip fracture and hospital admission, when the patients were in a estrange environment, in pain and anxious and were being treated with analgesic and sedating drugs. Thus, this assessment may not represent the true cognitive status of the patients before hospital admission; in turn, the second assessment was done after fracture stabilization, control of pain and beginning of rehabilitation. Resolution of the previous painful conditions may explain why most patients presented better scores in comparison to baseline. A population undergoing elective surgery would be a more appropriate setting to check the relationship between rCSO_2_ and cognitive decline.

Finally, 3 (10.3%) subjects died in the B group and none in LB group. The reported rate of death in this particular population is 5.3% [2], and the observation is consistent with these figures, considering the small sample size of our study that does not have power enough as to detect statistically significant differences in mortality.

## Conclusions

This prospective, randomized, clinical trial in very elderly patients could not show differences in cerebral oxygenation between B and LB during spinal anesthesia, but lower sensory and motor blockade was seen with LB than with B, and a trend to lower MAP and SPB was observed for B. While no differences were observed in cognitive impairment measured by the SPMSQ between treatment groups, postoperative neurological complications as reported by the physician were more frequent in B. An increased risk for intraoperative cerebral oxygen desaturation was observed for patients with lower trough intraoperative MAP and lower baseline rCSO_2_ values in the right hemisphere, and neurological complications were associated to lower cognitive status at the preoperative assessment. Finally, rCSO_2_ was not found as a predictive factor for cognitive impairment or late neurological complications.

## Abbreviations

ASA: American Society of Anesthesiologists
AUC: Area under the curve
B: Bupivacaine
HR: Heart rate
LB: Levobupivacaine
MAP: Mean arterial pressure
MB: Motor block
NC: Neurological complication
O2 Sat: O2 saturation
rCSO2: Regional cerebral oxygen saturation
SB: Sensory block
SD: Standard deviation
SPB: Systolic blood pressure
SPMSQ: Short Portable Mental Status Questionnaire
